# Lithium carbonate and coenzyme Q10 reduce cell death in a cell model of Machado-Joseph disease

**DOI:** 10.1590/1414-431X20165805

**Published:** 2016-11-21

**Authors:** C.M. Lopes-Ramos, T.C. Pereira, D.B. Dogini, R. Gilioli, I. Lopes-Cendes

**Affiliations:** 1 Departamento de Genética Médica, Faculdade de Ciências Médicas, Universidade Estadual de Campinas, Campinas, SP, Brasil; 2 Departamento de Biologia, Faculdade de Filosofia, Ciências e Letras de Ribeirão Preto, Universidade de São Paulo, Ribeirão Preto, SP, Brasil; 3 Centro Multidisciplinar para Investigação Biológica, Universidade Estadual de Campinas, Campinas, SP, Brasil

**Keywords:** Machado-Joseph disease, Spinocerebellar ataxia type 3, Lithium carbonate, Coenzyme Q10, Drug treatment

## Abstract

Machado-Joseph disease (MJD) or spinocerebellar ataxia type 3 (SCA3) is an autosomal dominant neurodegenerative disorder caused by expansion of the polyglutamine domain of the ataxin-3 (ATX3) protein. MJD/SCA3 is the most frequent autosomal dominant ataxia in many countries. The mechanism underlying MJD/SCA3 is thought to be mainly related to protein misfolding and aggregation leading to neuronal dysfunction followed by cell death. Currently, there are no effective treatments for patients with MJD/SCA3. Here, we report on the potential use of lithium carbonate and coenzyme Q10 to reduce cell death caused by the expanded ATX3 in cell culture. Cell viability and apoptosis were evaluated by MTT assay and by flow cytometry after staining with annexin V-FITC/propidium iodide. Treatment with lithium carbonate and coenzyme Q10 led to a significant increase in viability of cells expressing expanded ATX3 (Q84). In addition, we found that the increase in cell viability resulted from a significant reduction in the proportion of apoptotic cells. Furthermore, there was a significant change in the expanded ATX3 monomer/aggregate ratio after lithium carbonate and coenzyme Q10 treatment, with an increase in the monomer fraction and decrease in aggregates. The safety and tolerance of both drugs are well established; thus, our results indicate that lithium carbonate and coenzyme Q10 are good candidates for further *in vivo* therapeutic trials.

## Introduction

Machado-Joseph disease (MJD), also known as spinocerebellar ataxia type 3 (SCA3), is an autosomal dominantly inherited neurodegenerative disorder. It is caused by CAG triplet repeat expansions that encode an expanded polyglutamine (polyQ) tract in the disease-related protein, ataxin-3 (ATX3). Other members of this group include Huntington’s disease, SCA 1, 2, 6, 7, and 17, dentatorubral-pallidoluysian atrophy, and spinobulbar muscular atrophy. MJD/SCA3 is the most frequent type of autosomal dominant spinocerebellar ataxia in many countries, representing 15 to 45% of all SCAs (1,2). Clinical manifestations include ataxia, ophthalmoplegia, pyramidal signs, basal ganglia symptoms, and peripheral neuropathy (3,4).

MJD/SCA3 is characterized by an expansion of the polyQ tract near the C-terminus of the *MJD-1* gene product, ATX3 (5). Expanded polyQ tracts likely lead to protein misfolding, neuronal dysfunction and death. Although ATX3 is ubiquitously expressed, degeneration occurs preferentially in brain regions such as the substantia *nigra*, motor cranial nuclei, and dentate nucleus of the cerebellum (4). An abnormal accumulation of misfolded expanded ATX3, along with molecular chaperones, transcription factors or co-activators, and the components of the proteasome, forms highly ubiquitinated neuronal inclusions, which constitute a pathological hallmark of MJD/SCA3, as in other polyglutamine diseases (5,6).

Expanded ATX3 tends to accumulate in the cell nucleus and several studies suggest that these accumulating proteins cause transcription dysregulation through aberrant protein-protein interactions (1). It has been demonstrated that ATX3 sequesters the basal transcription factor TATA-binding protein and the transcriptional co-activator CREB-binding protein in the nuclear inclusions in cell-based models and in disease tissue. Furthermore, microarray gene expression profiling in MJD/SCA3 transgenic mice revealed that expanded ATX3 may cause cerebellar dysfunction and ataxia by disrupting the normal pattern of gene transcription (7). To date, there is no specific treatment for the neurodegeneration affecting patients with MJD/SCA3 (8).

Here, we report on the potential use of lithium carbonate and coenzyme Q10 (CoQ10) to reduce the cell death caused by the expanded ATX3 in a cultured cell model. We focused on two substances that are nontoxic and can be administered safely to patients.

## Material and Methods

### Cell culture, transfection and drug treatment

PC12 cells were grown in Dulbecco’s modified Eagle’s medium (DMEM, Gibco, USA) supplemented with 100 μg/mL penicillin/streptomycin, 2 mM L-glutamine, 10% fetal bovine serum and maintained at 37°C, 5% CO_2_ in a humidified incubator.

The expression vector pcDNA encoding human full-length ATX3 was used for transfection. Constructs of normal ATX3 (pcDNA3-myc-ATX3-Q28) and expanded ATX3 (pcDNA3-myc-ATX3-Q84) were previously reported (7), and were kindly provided by Dr. Henry Paulson, University of Michigan, Ann Arbor, MI, USA.

For transient transfections, cells were seeded on 6-well plates and grown to 60-80% confluence for 16 h. For each well, cells were exposed for 5 h to a mixture of 10 µL LipofectAMINE 2000 Reagent™ (Invitrogen, USA) and 2 μg of plasmid DNA in OptiMEM (Gibco, USA). Then, the culture medium was replaced by complete medium containing distinct drugs. Cells were incubated for another 48 h and examined. Drug treatments included 2.5, 5 or 7.5 mM lithium carbonate (Acros Organics™, Belgium) and 10, 30, or 90 μM coenzyme Q10 (Solgar™, USA).

### Cell viability and apoptosis

Cell viability was evaluated by the MTT, 3-(4,5-dimethylthiazole-2-yl)-2,5-diphenyl tetrazolium assay. After 48 h of drug treatment, the medium was replaced, MTT was added to the DMEM (0.3 mg/mL) followed by a 4-h incubation. Next, cells were washed with PBS, dye crystals were dissolved in DMSO and the absorbance measured at 570 nm.

Apoptotic cells were detected using the ApoTarget™ Annexin-V FITC Apoptosis Kit (Invitrogen), which employs a fluorescein-labeled Annexin-V (Annexin-V FITC) in concert with propidium iodide (PI), following manufacturer’s recommendation. Samples were analyzed on a FACScalibur flow cytometer (Becton Dickinson, USA). For each sample, a minimum of 10,000 events were collected and analyzed using CELLQuest software (Becton Dickinson). The PI results were used to exclude necrotic cells from the evaluation of apoptosis.

### Western blot

Protein was extracted with TRIzol reagent (Invitrogen, USA). Samples were run on discontinuous 10% SDS-polyacrylamide gels (PAGE) and both the stacking and separating portions of the gel were blotted onto Hybond-C Extra nitrocellulose membranes (GE Healthcare, USA). Blots were incubated with affinity-purified ATX3 antiserum (1:1,000, kindly provided by Dr. H. Paulson), followed by alkaline phosphatase-conjugated goat anti-rabbit IgG (1:20,000, Sigma, USA).

Aggregates are insoluble in SDS-PAGE and, therefore, remain in the stacking portion of the gel. ATX3 monomer/aggregate ratios were calculated based on band densitometry using ImageJ software.

### Statistical analysis

Results are reported as the means±SE value of 3 experiments. The Kruskal-Wallis test followed by the Student-Newman-Keuls test was performed using BioEstat 5.0 software (Brazil) to determine whether differences were statistically significant (P<0.05).

## Results

**Figure 1:**
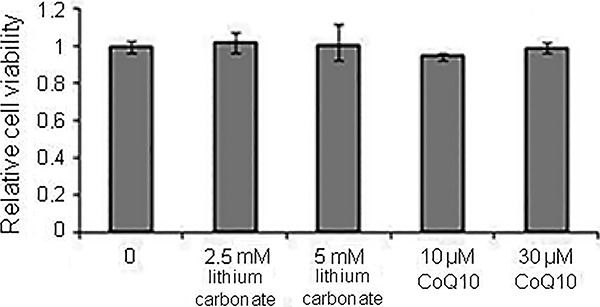
PC12 cells transfected with full-length normal ATX3 (Q28) and treated with different concentrations of lithium carbonate or coenzyme Q10 (CoQ10). Drug effects on cell viability were analyzed after 48 h. Relative cell viability was measured by the MTT assay. All experiments were performed in triplicate and data are reported as means±SE.

Transient expression of full-length expanded ATX3 (Q84) induced intracellular aggregate formation and cell death when compared with cells expressing constructs with normal ATX3 (Q28), as previously demonstrated (7,9–12). We evaluated the effects of two drugs on cell cultures expressing ATX3 constructs: lithium carbonate and CoQ10. Drug effects were initially evaluated in cell cultures transfected with normal ATX3 (Q28) construct for both drugs in different doses. We used this experiment as a control for any possible unspecific effects of the drugs on cell viability and proliferation. As expected, drug treatment did not alter viability of cells expressing normal ATX3 (Q28) (Figure 1).

### Lithium carbonate treatment

**Figure 2:**
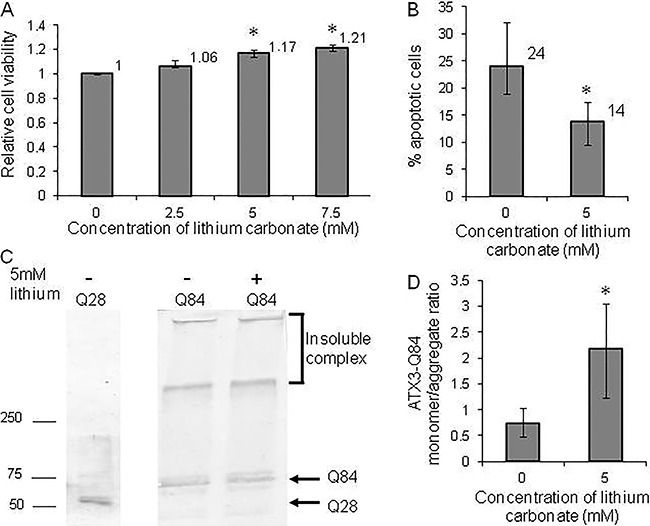
PC12 cells transfected with full-length expanded ATX3 (Q84), treated with different concentrations of lithium carbonate and analyzed after 48 h. *A*, Relative cell viability by the MTT assay. *B*, Percentage of apoptotic cells analyzed by flow cytometry after Annexin V and propidium iodide staining. *C*, ATX3-Q84 monomer/aggregate ratio analyzed by western blotting. *D*, Band densitometry was calculated with ImageJ software, and data of monomer/aggregate ratio were plotted in a graph. All experiments were performed in triplicates and data are reported as means±SE. *P<0.05, Kruskal-Wallis test.

Cells were treated with three concentrations of lithium carbonate, and cell viability was initially evaluated by the MTT assay. In cell cultures expressing the expanded ATX3 (Q84), we observed a relative increase in cell viability after treatment with a 5 mM initial lithium carbonate concentration. The effect was dose dependent: a 17 and 21% increase in cell viability was observed after treatment with 5 and 7.5 mM lithium carbonate, respectively (P=0.0117 and P=0.0046; Figure 2A). In contrast, no effect on cell viability was observed with the lowest concentration of lithium carbonate tested (2.5 mM). These results were confirmed by flow cytometry analysis after Annexin V/PI double staining. Although in cell cultures expressing ATX3-Q84 there were more apoptotic cells (compared to ATX3-Q28 cells), lithium treatment significantly reduced the percentage of apoptotic cells from 24 to 13.7% as demonstrated by flow cytometry analysis (P*=*0.0495; Figure 2B).

Western blot was chosen to evaluate a possible modification in the ATX3 monomer/aggregate ratio because ATX3-Q84 forms insoluble high molecular weight complexes at the top of stacking SDS gels. Indeed, we found that the monomer/aggregate ratio changed significantly after lithium treatment (P*=*0.0495), with an increase in the monomer fraction and decrease in the aggregates (Figure 2C and D).

### Coenzyme Q10 treatment (CoQ10)

**Figure 3:**
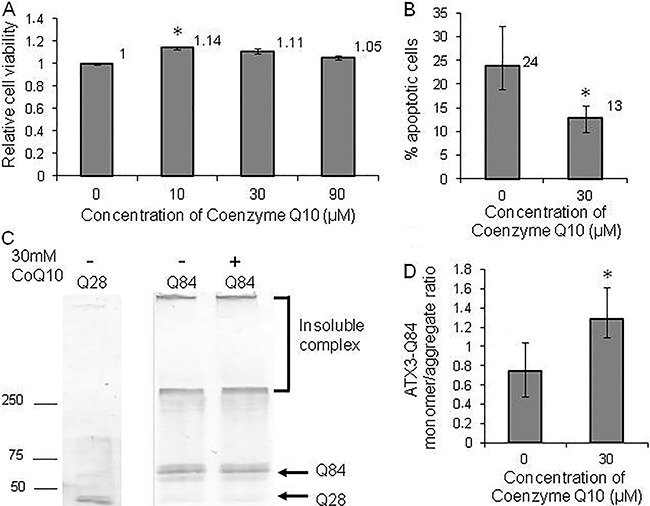
PC12 cells transfected with full-length expanded ATX3 (Q84), treated with different concentrations of coenzyme Q10 (CoQ10) and analyzed after 48 h. *A*, Relative cell viability was measured by the MTT assay. *B*, Percentage of apoptotic cells analyzed by flow cytometry after Annexin V and propidium iodide staining. *C*, ATX3-Q84 monomer/aggregate ratio analyzed by western blotting. *D*, Band densitometry was calculated with ImageJ software and the data of monomer/aggregate ratio were plotted in a graph. All experiments were performed in triplicate and data are reported as means±SE. *P<0.05, Kruskal-Wallis test.

Relative cell viability, measured by the MTT assay, increased by 14% after 10 μM of CoQ10 treatment (P=0.0357; Figure 3A). A greater concentration of CoQ10 (30 μM) increased cell viability by 11%, but not significantly (P=0.715; Figure 3A). In addition, we observed a 10% reduction in apoptotic cells by flow cytometry analysis after Annexin V/PI double staining (P=0.0339; Figure 3B). Furthermore, CoQ10 treatment significantly changed the ATX3-Q84 monomer/aggregate ratio (P=0.0495), with a relative increase in the monomer fraction and decrease in the aggregates (Figure 3C and 3D).

## Discussion

Strategies that decrease the cytotoxicity associated with expanded ATX3 *in vitro* models represent important therapeutic approaches. The activation of mitochondrial apoptotic pathways followed by increased mitochondrial DNA damage has been reported in cellular models of MJD, establishing a mitochondrial role in MJD pathology (13). However, we did not analyze the effects on mitochondrial function upon treatment with coQ10 and lithium in this phase of our study, but rather focused on the potential effects of these two drugs on ATX3-Q84-induced cell death.

One important issue that should be addressed when using the strategy we describe is the possibility that the results of increased cell viability be confounded by increased cell proliferation induced by the drugs tested. We addressed this issue by using cell cultures expressing ATX3-Q28 (normal ATX3 constructs) as controls and demonstrating that there was no variation in baseline measures in any of the experiments performed. These results indicate that the drugs did not induce cell proliferation because there was no variation in cell viability in cultures expressing ATX3-Q28 when subjected to the different treatments.

### Lithium carbonate treatment

Lithium carbonate significantly increased cell viability in 17 to 21% of cells expressing ATX3-Q84, as measured by the MTT assay, depending on the dose used. In addition, flow cytometry allowed us to specifically evaluate the percentage of apoptotic cells stained only for Annexin V. In cell cultures expressing ATX3-Q84, we found a 10% reduction in apoptotic cells after lithium treatment.

Lithium carbonate has been reported to have beneficial effects in different models of Huntington’s disease (HD) and SCA1. Lithium reduced the toxicity induced by cells expressing exon 1 of the *HD* gene with 74 CAGs in neuronal and non-neuronal cell lines (14). Chronic treatment with lithium caused a significant improvement in the rotarod performance of HD transgenic mice (R6/2 line) expressing exon 1 of the *HD* gene with approximately 150 CAGs (15). Furthermore, lithium treatment of SCA1 animal models expressing full-length ATX1 with 154 glutamines improved motor dysfunction and cognitive impairment (16).

On the other hand, a recent study showed that the treatment of a MJD transgenic mouse model (CMVMJD135) with a conjugation of lithium chloride and CCI-779 (Temsirolimus) at a nontoxic concentration known to induce autophagy was deleterious to both wild-type and transgenic animals (17). In another study performed by this same group, results that did not support lithium chronic treatment as a promising strategy for the treatment of MJD were reported (18).

We found a significant change in the ratio of monomer/aggregate of ATX3-Q84 after lithium treatment, with an increase in the monomer fraction and decrease in the aggregates. Lithium was reported to reduce the proportion of cells with aggregates in a HD cell model (14). Although aggregates represent a pathological hallmark of polyglutamine diseases, their association with neurodegeneration and precise role in disease pathogenesis remain unclear. Aggregate frequency and cytotoxicity induced by truncated expanded ATX3 were reduced after cell treatment with chemical chaperones (19). In addition, on immunoblot analyses, there was a decrease in insoluble complexes in the stacking gel with an increase in the monomeric protein; this was also reported for transgenic flies co-expressing truncated expanded ATX3 and molecular chaperones (20).

Lithium neuroprotective effects have been previously documented; however, its mechanism of action has not been fully established. Lithium may act through expression modulation of several genes such as *T*P*53*, *Bax*, *Bcl-2* and *GSK-3* (21,22). Over-expression of glycogen synthase kinase-3 (GSK-3) potentiates apoptosis in neuroblastoma cells, while GSK-3 inhibitors, such as lithium, protect cells against apoptosis (14,22,23). Since expanded ATX3 may disrupt normal gene transcription patterns (7), lithium may act as a protective factor by modulating gene expression.

The reduction in cell death induced by treatment with lithium carbonate in cells expressing expanded ATX3 demonstrated in our study is further evidence of the therapeutic potential of lithium in polyglutamine diseases, as demonstrated in HD and SCA1 models. Furthermore, since this drug has been used in patients for over 50 years, its therapeutic potential and toxic effects are well known (24). However, one should be aware that among the side effects of lithium treatment are tremors and lack of coordination, especially when the dose is not well monitored (25). These side effects may be an additional risk in patients with cerebellar dysfunction; therefore, Watase et al. (16) evaluated the occurrence of tremors in mice during treatment with lithium and found no increase of these events as compared to untreated animals.

Recent studies reporting trials with lithium treatment in patients with MJD/SCA3 had somewhat limited results (26,27), but there were some suggestions that lithium treatment could be beneficial to patients in the early stages of the disease and to specific symptoms such as cerebellar dysfunction (27).

### Coenzyme Q10 treatment

Our results showed that CoQ10 treatment protected against ATX3-Q84-induced death in PC12 cells, increasing cell viability. There was an 11% reduction in apoptotic cells, with no induction of cell proliferation as a confounding factor. In addition, we also found a significant change in the ratio of monomer/aggregate of ATX3-Q84 after CoQ10 treatment. Aggregate reduction was also observed in transgenic mouse models for HD treated with CoQ10 (28,29). In a tolerance study, CoQ10 was well tolerated and safe in patients at doses as high as 3000 mg/day, but the plasma CoQ10 level reaches a plateau at the dose of 2,400 mg/day (30).

There is some evidence of mitochondrial dysfunction and bioenergetic abnormalities in the pathogenesis of neurodegenerative diseases (31). Analyses of HD *post mortem* brain tissues showed impaired oxidative phosphorylation enzyme activity, decreased mitochondrial complex activity, and increased oxidative damage product 8-hydroxydeoxyguanosine (32). Additionally, a MJD/SCA3 *in vitro* model demonstrated that expanded ATX3 impairs the cell’s ability to respond to stress, alters antioxidant enzyme activities, and promotes mitochondrial DNA damage, which may lead to mitochondrial dysfunction (13). Thus, compounds that enhance cellular and mitochondrial bioenergetics are interesting candidates for treating MJD/SCA3. In this work, we studied CoQ10, a cofactor of the electron transport chain and a potent antioxidant (33).

Several *in vitro* models have previously demonstrated the neuroprotective effects of CoQ10. In neuronal models of oxidative stress, CoQ10 pretreatment preserves mitochondrial membrane potential and reduces the generation of reactive oxygen species (34). In cultured neurons from HD transgenic mice, cell death is reduced after CoQ10 treatment (35). Other studies showed that oral administration of CoQ10 in a transgenic HD mouse model extends survival, improves motor performance and reduces brain atrophy (28,29,36).

The elevated levels of lactate in the cerebral cortex and basal ganglia of HD patients were reduced after CoQ10 treatment (37). These results suggest that CoQ10 has metabolic effects in cerebral tissue. The Huntington's Study Group (38) performed the CARE-HD trial using CoQ10 alone or in combination with remacemide and demonstrated the safety and tolerance of these drugs. In addition, CoQ10 reduced the trend toward cognitive and functional decline. Moreover, several clinical trials for Parkinson’s disease, Alzheimer disease, amyotrophic lateral sclerosis and Friedreich ataxia demonstrated the potential neuroprotective effects of CoQ10 (39).

Besides being a natural compound, safe and well tolerated with few side effects (30,40), CoQ10 is able to cross the brain barrier, as demonstrated in animal studies. CoQ10 oral administration in HD mice increased the levels of CoQ10 in the brain of these animals (29). Thus, our results together with evidence from the literature point to the potential of this compound for treating patients with MJD/SCA3. To our knowledge, there have been no clinical trials with CoQ10 in patients with MJD/SCA3.

In conclusion, we demonstrated that lithium carbonate and CoQ10 reduced apoptosis induced by expanded ATX3 (Q84). Currently, there are no effective treatments for patients with MJD/SCA3, hence, the importance of studying compounds capable of reducing the disease’s cytotoxic effects. Safety and tolerance of both drugs are well established; thus our results indicate that lithium carbonate and CoQ10 can be putative candidates for further *in vivo* therapeutic trials.
